# Editorial: Insights into solar radiation: therapeutic targets and innovative products

**DOI:** 10.3389/fphar.2026.1925670

**Published:** 2026-07-17

**Authors:** Fernanda Maria Pinto Vilela, Laura A. Calvo-Castro, Yris Maria Fonseca-Bazzo

**Affiliations:** 1 Laboratory of Nanostructured Systems Development, Department of Pharmaceutical Science, Faculty of Pharmacy, Federal University of Juiz de Fora, Juiz de Fora, Minas Gerais, Brazil; 2 Centro de Investigación en Biotecnología, Instituto Tecnológico de Costa Rica, Cartago, Costa Rica; 3 Laboratory of Natural Products, Faculty of Health Sciences, University of Brasilia, Brasília, Brazil

**Keywords:** antioxidant, dermatology, radiation, Skin, therapeutic targets

Solar radiation has accompanied human evolution since the beginning, providing warmth, enabling vision, and sustaining life through photosynthesis (Janssen et al., 2014). However, the same ultraviolet (UV) photons that trigger vitamin D synthesis can also induce DNA damage (Neville et al., 2021); the same infrared wavelengths that promote tissue repair can accelerate photoaging (Applegate et al., 2000; Schieke et al., 2003). This Research Topic brings together fundamental contributions that explore the duality of solar radiation, proposing new therapeutic targets and innovative agents for skin protection and treatment.

This Research Topic, Insights into Solar Radiation: Therapeutic targets and Innovative products, was conceived to bridge a persistent gap: while photobiology has traditionally focused on preventing harm (e.g., sunscreens, skin cancer, and immunosuppression), recent advances demand a parallel focus on therapeutic exploitation. How can we harness specific spectral bands to treat inflammatory skin diseases, chronic wounds, or even mood disorders? Which molecular pathways activated by solar radiation can be selectively targeted for drug development? What innovative formulations—from topical photoprotectors that also deliver active ingredients to wearable light sensors—are emerging at the intersection of dermatology, optics, and bioengineering?

We received contributions that collectively addressed these questions from multiple angles.

The Review by Fan et al. revisited ultraviolet radiation (UVR) as a “double-edged regulator”. The authors discussed how UVR, while therapeutic for treating vitiligo by stimulating melanocyte stem cell differentiation and melanogenesis via the *Wnt/β-catenin* pathway, also induces genotoxic stress and genomic instability, thereby predisposing individuals to melanoma. The review emphasizes the importance of combining phototherapy with *JAK* inhibitors to optimize repigmentation and stabilize the immune response, setting new standards for clinical management (Fan et al.).

Two Original Research articles focused on innovative molecules that overcome the limitations of classic antioxidants.

Piffaut et al. investigated the prevention of pigmentation induced by high-energy visible (HEV) light. In two randomized controlled trials, the authors demonstrated that 2- mercaptonicotinoyl glycine (2-MNG) is effective in significantly reducing HEV-induced hyperpigmentation. In contrast, ascorbic acid (vitamin C), despite being a potent antioxidant, showed no efficacy against this specific spectrum. This opens the door to transparent and effective photoprotection solutions for non-covering and non-colored products (Piffaut et al.).

Pasuch Gluzezak et al. presented Trz-HBH3, a novel resveratrol analog obtained through molecular triplication. This compound demonstrated high photostability and the ability to reduce UVA-induced reactive oxygen species (ROS) by up to 69.5% in advanced 3D bioprinted endothelialized human skin models, reinforcing the applicability of bioprinted skin as an advanced *in vitro* platform for investigating UV-induced skin responses (Pasuch Gluzezak et al.).

The Mini Review by Dong et al. focused on plant-derived exosome-like nanovesicles (PELNs). These structures offer a biocompatible, low-toxicity alternative for drug delivery. The study reviewed how PELNs from lavender, aloe vera, and grapes can attenuate UVB-induced photoaging by regulating pathways involved in DNA repair and *collagen* synthesis, representing a promising frontier for “medical cosmetics”. The authors discussed the current challenges and future directions associated with the development and application of PELNs as innovative anti-photoaging agents (Dong et al.).

We hope this Research Topic stimulates not only further mechanistic studies but also dialogue between photobiologists, dermatologists, regulatory scientists, and industry partners. Solar radiation will remain a constant companion to life on Earth. Our task is to understand it deeply enough to transform its risks into opportunities, and its energy into targeted healing.
